# Scheduled feeding improves sleep in a mouse model of Huntington’s disease

**DOI:** 10.3389/fnins.2024.1427125

**Published:** 2024-08-05

**Authors:** Emily Chiem, Kevin Zhao, Derek Dell’Angelica, Cristina A. Ghiani, Ketema N. Paul, Christopher S. Colwell

**Affiliations:** ^1^Department of Integrative Biology and Physiology, University of California Los Angeles, Los Angeles, CA, United States; ^2^Molecular, Cellular, Integrative Physiology Program, University of California Los Angeles, Los Angeles, CA, United States; ^3^Department of Psychiatry and Biobehavioral Sciences, University of California Los Angeles, Los Angeles, CA, United States; ^4^Department of Pathology and Laboratory Medicine, University of California Los Angeles, Los Angeles, CA, United States

**Keywords:** BACHD, EEG, Huntington’s disease, sleep, time restricted feeding, sex

## Abstract

Sleep disturbances are common features of neurodegenerative disorders including Huntington’s disease (HD). Sleep and circadian disruptions are recapitulated in animal models, providing the opportunity to evaluate the effectiveness of circadian interventions as countermeasures for neurodegenerative disease. For instance, time restricted feeding (TRF) successfully improved activity rhythms, sleep behavior and motor performance in mouse models of HD. Seeking to determine if these benefits extend to physiological measures of sleep, electroencephalography (EEG) was used to measure sleep/wake states and polysomnographic patterns in male and female wild-type (WT) and bacterial artificial chromosome transgenic (BACHD) adult mice, under TRF and *ad lib* feeding (ALF). Our findings show that male, but not female, BACHD mice exhibited significant changes in the temporal patterning of wake and non-rapid eye movement (NREM) sleep. The TRF intervention reduced the inappropriate early morning activity by increasing NREM sleep in the male BACHD mice. In addition, the scheduled feeding reduced sleep fragmentation (# bouts) in the male BACHD mice. The phase of the rhythm in rapid-eye movement (REM) sleep was significantly altered by the scheduled feeding in a sex-dependent manner. The treatment did impact the power spectral curves during the day in male but not female mice regardless of the genotype. Sleep homeostasis, as measured by the response to six hours of gentle handling, was not altered by the diet. Thus, TRF improves the temporal patterning and fragmentation of NREM sleep without impacting sleep homeostasis. This work adds critical support to the view that sleep is a modifiable risk factor in neurodegenerative diseases.

## Introduction

Sleep disturbance is a common feature of neurodegenerative diseases, such as Huntington’s disease (HD) (Colwell, 2021; Voysey et al., 2021). Huntington’s disease is caused by an abnormal CAG repeat expansion within the *huntingtin* (*Htt*) gene, which leads to widespread physiological disruption (Tabrizi et al., 2020). Sleep questionnaires find that HD patients commonly experience insomnia, daytime sleepiness, delayed sleep onset, and frequent nighttime awakenings (Goodman et al., 2011; Herzog-Krzywoszanska and Krzywoszanski, 2019; Tanigaki et al., 2020; Ogilvie et al., 2021). Smaller scale polysomnography (PSG) studies have also uncovered delayed sleep onset, increased sleep fragmentation as well as decreased slow-wave sleep (Arnulf et al., 2008; Cuturic et al., 2009; Goodman et al., 2011; Lazar et al., 2015). Broadly, these sleep/wake cycle disturbances described in the HD carriers are recapitulated in mouse models. The R6/2 mouse model of HD exhibits increased activity during the day and sleep fragmentation (Morton et al., 2005; Kantor et al., 2013) and the Q175 model has been shown to display increased wakefulness and decreased NREM sleep during the light phase (Loh et al., 2013; Fisher et al., 2016). In addition, the BACHD mouse model of HD exhibits sleep/wake architecture disruptions (Kudo et al., 2011), which are sex dependent (Chiem et al., 2024). Overall, there is comprehensive evidence of abnormal electroencephalography (EEG)-defined sleep architecture in mouse (Fisher et al., 2013; Kantor et al., 2013; Lebreton et al., 2015; Fisher et al., 2016) and sheep (Schneider et al., 2021; Vas et al., 2021) models of HD. These studies consistently found an early and progressive deterioration of both sleep architecture and behavior.

Diurnal rhythms in the sleep/wake cycle are generated, in part, by the circadian timing system with the master clock residing in the suprachiasmatic nucleus (SCN) in the hypothalamus. Signs of extensive degeneration and neuronal loss in the SCN have been reported in postmortem brain from HD individuals (van Wamelen et al., 2014). Likewise, in mouse models, there are reductions in the expression of the neuropeptide vasoactive intestinal polypeptide (VIP) and its receptor VPAC2 within the SCN (Fahrenkrug et al., 2007; Kuljis et al., 2016) as well as in the neural activity rhythms that are the hallmark of SCN function (Kudo et al., 2011; Kuljis et al., 2016, 2018). Therefore, in the case of HD and perhaps other neurodegenerative models, the ideal intervention could be effective even with a compromised SCN. For instance, the daily feed/fast cycle is a powerful regulator of the circadian system that functions even when the SCN is damaged (Stephan, 1983, 1989; Angeles-Castellanos et al., 2010; Mistlberger, 2011). In early work with the R6/2 model, food entrainment aided in the maintenance of body temperature rhythms and improved locomotor behavior (Maywood et al., 2010; Skillings et al., 2014). A time-restricted feeding (TRF) protocol has been shown to improve locomotor activity, sleep behavioral patterns, and heart rate variability in Q175 mice (Wang et al., 2018) and BACHD mice (Whittaker et al., 2018). Since this prior work did not examine the possible impact of TRF on EEG-defined sleep, in the present study, we utilized the BACHD mouse model of HD to examine the impact of TRF on sleep/wake architecture, EEG spectral power, and the homeostatic response to sleep deprivation.

## Methods

### Animals

All the experimental protocols used to collect the data for the present report were approved by the UCLA Animal Research Committee and followed the guidelines and recommendations for animal use and welfare set by the UCLA Division of Laboratory Animal Medicine and National Institutes of Health. The BACHD mouse model of HD contains a human mutant *Htt* gene encoding 97 glutamine repeats (Gray et al., 2008). BACHD females backcrossed on a C57BL/6 J background were bred in-house with C57BL/6 J (WT) males from the Jackson Laboratory to obtain male and female offspring, either WT or heterozygous for the BACHD transgene. The WT littermates were used as controls in this study. There were four groups of mice used in this study including both male (WT, *n* = 12; BACHD, *n* = 12) and female (WT, *n* = 12; BACHD, *n* = 12) mice in order to further our knowledge on the sex difference. In our colony, the average body weight of these groups at 3 months is: WT Male, 26.3 ± 0.6; WT Female, 21.7 ± 0.4; BACHD Male, 27.3 ± 0.6; BACHD Female, 26.2 ± 0.7. Some of the recordings did not generate clear signals and were excluded from analysis. Animals were group housed (4 per cage), and entrained to a 12:12 LD cycle, in sound-proof, humidity-controlled chambers until experimentation began.

### Surgery

All animals were surgically implanted just prior to 3 months of age with EEG and electromyograph (EMG) electrodes for polysomnography recordings. A prefabricated headmount (Pinnacle Technologies, Lawrence, KS) was used to position three stainless-steel epidural screw electrodes. The first electrode (frontal- located over the frontal cortex) was placed 1.5 mm anterior to bregma and 1.5 mm lateral to the central suture. The second two electrodes (interparietal- located over the visual cortex and common reference) were placed 2.5 mm posterior to bregma and 1.5 mm on either side of the central suture. The resulting two leads (frontal-interparietal and interparietal-interparietal) were referenced contralaterally. A fourth screw served as ground. Silver epoxy was used to aid electrical continuity between the screw electrode and headmount. Stainless-steel teflon-coated wires were inserted bilaterally into the nuchal muscle to record EMG activity. The headmount was secured to the skull with dental acrylic.

### EEG/EMG recording

Mice were placed in sound-proof sleep-recording chambers and connected to a lightweight tether attached to a low-resistance commutator mounted over the cage (Pinnacle Technologies). The animals were allowed free range of movement throughout the cage while being tethered and given one week to acclimate to the tether and recording chambers. EEG and EMG recordings began at zeitgeber time (ZT) 0 (light onset) and continued for 24 h. Data acquisition was performed on a personal computer running Sirenia Acquisition software (Pinnacle Technologies), a software system specific to rodent polysomnographic recordings. EEG signals were low-pass filtered with a 40-Hz cutoff and collected continuously at a sampling rate of 400 Hz. After data collection, waveforms were scored by the same trained operator as wake (low-voltage, high-frequency EEG; high-amplitude EMG), NREM sleep (high voltage, mixed frequency EEG; low-amplitude EMG), or REM sleep (low-voltage EEG with a predominance of theta activity (5-7 Hz); low amplitude EMG). We did not use a camera in this recording set up. Recordings were scored in 10-s epochs as we have previously found that this resolution was sufficient for measuring diurnal rhythms (Ehlen et al., 2013). The operator was masked to the sex, genotype, and treatment of the mice.

### Signal analysis

Spectral analysis was performed on the frontal-interparietal lead. Power spectral analysis was performed by applying a fast Fourier transform (FFT) to raw EEG waveforms. Absolute power (μV^2^) was analyzed in 0.1 Hz bins across the entire power spectrum (0–40 Hz) using Sirenia Sleep Pro software (Pinnacle Technologies). Relative power in the delta (0.5–4 Hz), theta (5–7 Hz), alpha (8–12 Hz), beta (14–20 Hz), and gamma (20–40 Hz) bands was measured across the 24-h period. Relative NREM delta power during recovery (ZT6-24) was normalized to the average 24-h baseline NREM delta power for each animal. This analysis reflects the accumulation of delta power that occurs during wakefulness and the dissipation that occurs during NREM sleep, as delta power returns to baseline values. Sleep fragmentation was measured by the number of NREM sleep bouts and duration of N sleep bouts (sec).

### Total sleep deprivation

Immediately following a 24-h baseline recording, mice underwent 6-h of total sleep deprivation (SD) using a gentle-handling protocol, which includes cage tapping, introduction of novel objects, and delicate touching when mice displayed signs of sleep onset. SD began at the onset of the light phase in a 12 h:12 h LD cycle. Recordings continued for an 18-h of recovery opportunity following the period of forced wakefulness.

### Time restricted feeding (TRF)

Male and female WT and BACHD mice (3 months old) were exposed to one of two feeding regimen for 1 month: *ad libitum* feeding (ALF) or feeding restricted to 6 h during the middle of the active phase (ZT15-21). The three-month age point was chosen as the disruption in the locomotor activity rhythms just begins at this age (Kudo et al., 2011) and yet the mice are still physically robust and can handle surgery (Whittaker et al., 2018; Park et al., 2021). All mice had *ad lib* access to water. Mice were singly housed, and the bedding was changed twice a week as mice are coprophagic. For the first three weeks of TRF, mice were housed in cages with a custom-made programmable food hopper that controls food access. At the start of the fourth week of TRF, mice were connected to tethers and allowed one week to acclimate prior to EEG recordings. TRF was performed manually during this time, as the programmable food hoppers did not fit in the recording cages. Control mice were singly housed and given *ad lib* access to food and water.

### Statistical analysis

The data generated in this study were evaluated with either a two-way or three-way analysis of variance (ANOVA) to analyze the effects of two or three independent variables (factors: genotype, treatment and sex or time) on dependent variables measured by the EEG. We considered the main effects of each factor as well as any interaction between or among the factors. The waveforms under baseline or after SD were analyzed by three-way with genotype, sex, and time as factors. The total time spent in each state (wake, REM and NREM sleep), the amplitude or phase of the rhythms, and response to SD were analyzed by two-way ANOVA with genotype and sex as factors. The Holm-Sidak multiple comparison test was used when appropriate. Between-group differences were determined significant if *p* < 0.05. The normal distribution of the data was tested with a Shapiro–Wilk test and the assumption of equal variance was tested with a Brown-Forsythe test.

In addition to the ANOVAs, a linear mixed effects (LME) model was used to examine the spectral data. Each of the two methods serves different purposes and has different assumptions. The LME offers particular advantages for repeated measures while better accounting for subject-specific variability and the correlated structure of the data. This model incorporates fixed effects (genotype, treatment, sex, and time) as well as helping to understand the extent to which variability in the outcome can be explained by the fixed and random effects. For the spectral curve analysis, we included spectral power values (Hz) as the dependent variable and fixed effects of genotype, time of day, treatment, sex, and frequency. Each animal was included as a random effect. The model specifications were as follows: power values ~ Genotype * Time * Treatment * Sex * Frequency + (1 | Animal ID). The LME analyses was conducted using the *lme4* package in R version 4.2.1 (Bates et al., 2015) while the ANOVAs were conducted using SigmaPlot (version 14.5; SYSTAT Software, San Jose, CA).

To evaluate the rhythmicity of individual animals, the wake/REM/NREM measures for 24-h were analyzed using a Cosinor analysis^1^ by Dr. R. Refinetti to determine goodness of fit (0–1.0) with 1 being the worst. This program measures the similarity between the cosine of the angle between the vectors of the empirical data and a fitted cosine wave. The amplitude of the diurnal cycle was also determined by the Cosinor analysis as half the distance between the peak and trough.

## Results

### Wake rhythms

**Figure 1:**
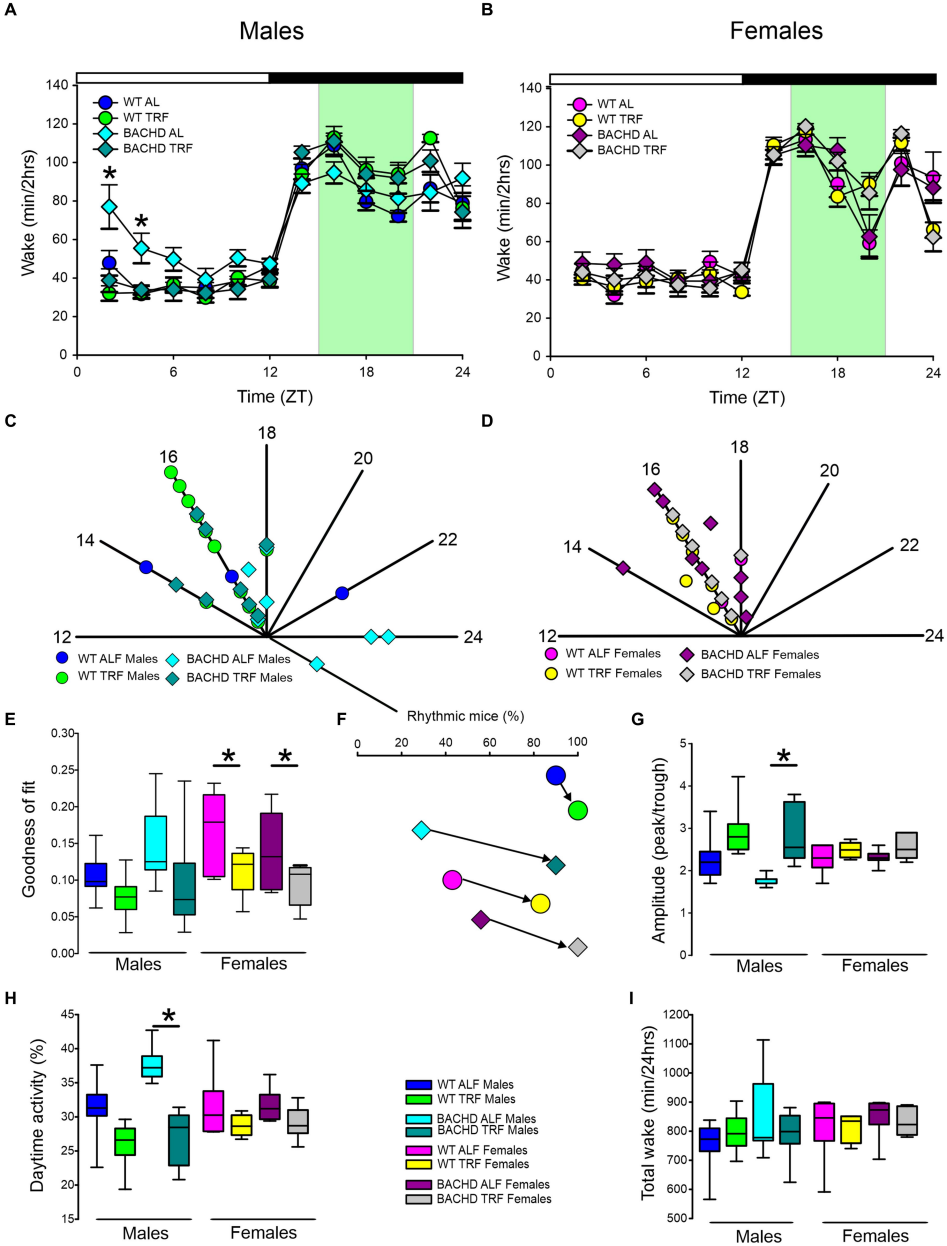
Temporal pattern of wake in BACHD and WT mice under TRF and ALF. EEG recordings were conducted over 24-h in undisturbed mice held in a 12 h:12 h light/dark (LD) cycle. Waveforms showing the daily rhythms of wake in male **(A)** and female **(B)** WT and BACHD mice plotted in 2-h bins. The BACHD males displayed increased wake during the lights-on (inactive) phase. This increase was corrected by the TRF intervention. Data are presented as the mean ± SEM (*n* = 6–9 animals/sex/genotype/experimental group). Time points in which significant differences (*p* < 0.05) were found between the groups using multiple comparisons procedures (Holm-Sidak method) are indicated by one asterisk. For this and the other figures, the timing of the light/dark cycle is shown by the bar at the top of the graph. The green-shaded area represents the time of scheduled feeding for the TRF treated groups. The circles represent the WT groups and the diamonds the BACHD groups. Polar display of the peak phase of the rhythms of wake in male **(C)** and female **(D)** WT and BACHD mice. The numbers on the axis represent zeitgeber time (ZT) with ZT 12 indicating the time of lights-off. The male BACHD on ALF exhibited higher variability in peak phase than the other groups. **(E)** The vector of the diurnal rhythms in wake were fit to a cosine wave and the “goodness of fit” was determined for each animal. The lower the number the better the fit. The median was calculated and is shown in the box plot. **(F)** The percentage of mice exhibiting a significant diurnal rhythm for each group. The lines with arrow connect each genotype and sex on ALF and TRF. This display shows that the scheduled feeding increases the number of mice with improved rhythms in wake. The amplitude of the rhythms **(G)** as well as the percentage (%) of wake during the day **(H)** and the total minutes of recorded wake **(I)** are displayed for each group. The TRF treatment significantly increased the amplitude of the male BACHD rhythm and reduced the percentage of daytime activity compared to BACHD mice on ALF. The box plots show the distribution of numerical data and skewness by displaying the data quartiles and median. *p < 0.05, Holm-Sidak multiple comparisons test.

In this study, we sought to use EEG measurements to test the hypothesis that TRF would impact daily rhythms of sleep/wake architecture and investigate the effects of sex on the response in WT and BACHD mice. First, we examined the rhythm in wake looking at 2-h bins across the 24 h cycle under ALF and TRF (Figures 1A,B). The waveforms were analyzed using a three-way ANOVA with genotype, treatment, and time as factors for both male and female mice, separately. Time was highly significant for both sexes, and the male mice also exhibited significant differences by genotype (Table 1). For both sexes, there was a significant interaction between treatment and time (Table 1). The elevated early morning wakefulness characteristic of the male BACHD was corrected by the TRF intervention (Figure 1A). There were no significant differences in the peak phase of the wake rhythms among groups (Figures 1C,D; Table 2). Still, the male, but not the female, BACHD exhibited a great deal of variability in phase (Table 3) compared to the WT, which was corrected by TRF. As measured by Cosinor analysis, the percentage of mice with a significant diurnal rhythm in wake and the amplitude of the rhythms were increased by the treatment, with the BACHD males showing the highest improvements elicited by TRF (Figures 1E,F; Tables 4, 5). We quantified the amplitude of day/night difference as a ratio of minutes of wake in the night over the minutes measured during the day. TRF increased the amplitude of the rhythms with the strongest and most significant effect in the male BACHD (Figure 1G; Table 2). The male BACHD mice exhibited more wake during the day (resting period) and less during the night (active period) and this aberration was corrected by TRF (Figure 1H). The total amount of wake did not vary with genotype, sex, or feeding schedule (Figure 1I; Table 2). Thus, as measured by EEG, TRF regularized the disrupted rhythm in the wake state in the male BACHD.

**Table 1:** Analysis of waveforms by three-way ANOVA with genotype, treatment, and time (2 h bins) as factors.

State	Sex	Genotype	Time (2 h bins)	Treatment	Interaction Treatment x Time
Wake	Males	***F***_**(1,419)**_ **= 5.272, *p* < 0.022**	***F***_**(11,419)**_ **= 109.8, *p* < 0.001**	*F*_(1,419)_ = 0.270, *p* = 0.604	***F***_**(11,419)**_ **= 6.892, *p* < 0.001**
	Females	*F*_(1,335)_ = 1.891, *p* = 0.170	***F***_**(11,335)**_ **= 117.3, *p* < 0.001**	*F*_(1,335)_ = 0.180, *p* = 0.668	***F***_**(11,335)**_ **= 5.029, *p* < 0.001**
REM	Males	*F*_(1,419)_ = 1.141, *p* = 0.286	***F***_**(11,419)**_ **= 77.93, *p* < 0.001**	***F***_**(1,419)**_ **= 5.559, *p* = 0.018**	*F*_(11,419)_ = 1.526, *p* = 0.120
	Females	***F***_**(1,335)**_ **= 6.264, *p* = 0.013**	***F***_**(11,335)**_ **= 125.6, *p* < 0.001**	*F*_(1,335)_ = 0.126, *p* = 0.722	***F***_**(11,335)**_ **= 2.654, *p* = 0.003**
NREM	Males	*F*_(1,419)_ = 3.332, *p* = 0.069	***F***_**(11,419)**_ **= 119.8, *p* < 0.001**	*F*_(1,419)_ = 1.225, *p* = 0.269	***F***_**(11,419)**_ **= 5.771, *p* < 0.001**
	Females	*F*_(1,335)_ = 0.049, *p* = 0.825	***F***_**(11,335)**_ **= 129.6, *p* < 0.001**	*F*_(1,335)_ = 7.201, *p* = 0.008	***F***_**(11,335)**_ **= 6.076, *p* < 0.001**

Interactions between treatment and time are reported. Degrees of freedom are reported within parentheses, alpha = 0.05. Bold type indicates statistical significance.

**Table 2:** Analysis of key parameters of each state (wake, REM, and NREM sleep) by three-way ANOVA with genotype, sex, and treatment as factors.

State		Genotype	Sex	Treatment
Wake	Amplitude	*F*_(1, 62)_ = 1.329, *p* = 0.254	***F***_**(1, 62)**_ **= 22.72, *p* < 0.001**	*F*_(1, 62)_ = 0.096, *p* = 0.757
	Phase	*F*_(1, 62)_ = 0.201, *p* = 0.656	*F*_(1, 62)_ = 0.955, *p* = 0.333	*F*_(1, 62)_ = 0.007, *p* = 0.932
	Day (%)	***F***_**(1, 62)**_ **= 5.364, *p* = 0.024**	***F***_**(1, 62)**_ **= 37.98, *p* < 0.001**	*F*_(1, 62)_ = 0.105, *p* = 0.748
	Total	*F*_(1, 62)_ = 2.656, *p* = 0.109	*F*_(1, 62)_ = 0.180, *p* = 0.673	*F*_(1, 62)_ = 2.112, *p* = 0.152
REM	Amplitude	*F*_(1, 56)_ = 0.089, *p* = 0.766	*F*_(1, 56)_ = 1.618, *p* = 0.209	*F*_(1, 56)_ = 0.444, *p* = 0.508
	Phase	*F*_(1, 61)_ = 0.314, *p* = 0.577	***F***_**(1, 61)**_ **= 7.808, *p* = 0.007**	***F***_**(1, 61)**_ **= 9.546, *p* = 0.003**
	Night (%)	*F*_(1, 61)_ = 1.160, *p* = 0.286	*F*_(1, 61)_ = 1.714, *p* = 0.196	*F*_(1, 61)_ = 0.154, *p* = 0.696
	Total	*F*_(1, 61)_ = 3.730, *p* = 0.059	*F*_(1, 61)_ = 1.366, *p* = 0.248	*F*_(1, 61)_ = 0.988, *p* = 0.322
NREM	Amplitude	*F*_(1, 61)_ = 1.335, *p* = 0.253	*F*_(1, 61)_ = 0.991, *p* = 0.324	*F*_(1, 61)_ = 0.162, *p* = 0.689
	Phase	***F***_**(1, 62)**_ **= 14.68, *p* < 0.001**	*F*_(1, 62)_ = 3.662, *p* = 0.061	***F***_**(1, 62)**_ **= 6.440, *p* = 0.014**
	Night (%)	*F*_(1, 62)_ = 0.569, *p* = 0.812	*F*_(1, 62)_ = 2.706, *p* = 0.106	*F*_(1, 62)_ = 1.475, *p* = 0.230
	Total	*F*_(1, 62)_ = 0.384, *p* = 0.538	*F*_(1, 62)_ = 3.554, *p* = 0.065	***F***_**(1, 62)**_ **= 5.970, *p* = 0.018**

Degrees of freedom are reported within parentheses, alpha = 0.05. Bold type indicates statistical significance.

**Table 3:** Confidence intervals of the peak phase of the EEG rhythms by genotype and sex.

State	Genotype	Sex	Treatment	Confidence interval
Wake	WT	Males	ALF	1.680
			TRF	0.601
		Females	ALF	0.857
			TRF	0.542
	BACHD	Male	ALF	**6.817**
			TRF	1.072
		Females	ALF	1.153
			TRF	0.699
REM sleep	WT	Male	ALF	1.883
			TRF	2.380
		Females	ALF	3.926
			TRF	2.550
	BACHD	Male	ALF	2.767
			TRF	1.182
		Females	ALF	1.427
			TRF	1.664
NREM sleep	WT	Male	ALF	1.559
			TRF	1.540
		Females	ALF	2.512
			TRF	1.084
	BACHD	Male	ALF	**2.767**
			TRF	1.537
		Females	ALF	**5.381**
			TRF	0.903

The BACHD males exhibited strikingly more variable measures for each state.

**Table 4:** Analysis of goodness of fit by three-way ANOVA with genotype, sex, and treatment as factors.

State	Genotype	Sex	Treatment
Wake	*F*_(1,62)_ = 0.382, *p* = 0.539	***F***_**(1,62)**_ **= 4.246, *p* = 0.044**	***F***_**(1,62)**_ **= 20.12, *p* < 0.001**
REM	*F*_(1,61)_ = 0.032, *p* = 0.858	*F*_(1,61)_ = 1.116, *p* = 0.298	***F***_**(1,62)**_ **= 8.123, *p* = 0.006**
NREM	*F*_(1,62)_ = 0.049, *p* = 0.825	***F***_**(1,62)**_ **= 9.428, *p* = 0.003**	***F***_**(1,62)**_ **= 20.611, *p* < 0.001**

No significant interactions between the three factors were detected. Degrees of freedom are reported within parentheses, alpha = 0.05. Bold type indicates statistical significance.

**Table 5:** Analysis of amplitude as measured by cosine analysis using three-way ANOVA with genotype, sex, and treatment as factors.

State	Genotype	Sex	Treatment
Wake	*F*_(1,62)_ = 3.043, *p* = 0.087	*F*_(1,62)_ = 1.092, *p* = 0.301	***F***_**(1,62)**_ **= 22.29, *p* < 0.001**
REM	*F*_(1,61)_ = 1.379, *p* = 0.242	*F*_(1,61)_ = 0.001, *p* = 0.981	*F*_(1,62)_ = 1.379, *p* = 0.245
NREM	*F*_(1,62)_ = 0.425, *p* = 0.517	*F*_(1,62)_ = 2.776, *p* = 0.101	***F***_**(1,62)**_ **= 20.17, *p* < 0.001**

No significant interactions among the three factors were detected. Degrees of freedom are reported within parentheses, alpha = 0.05. Bold type indicates statistical significance.

### REM sleep rhythms

**Figure 2:**
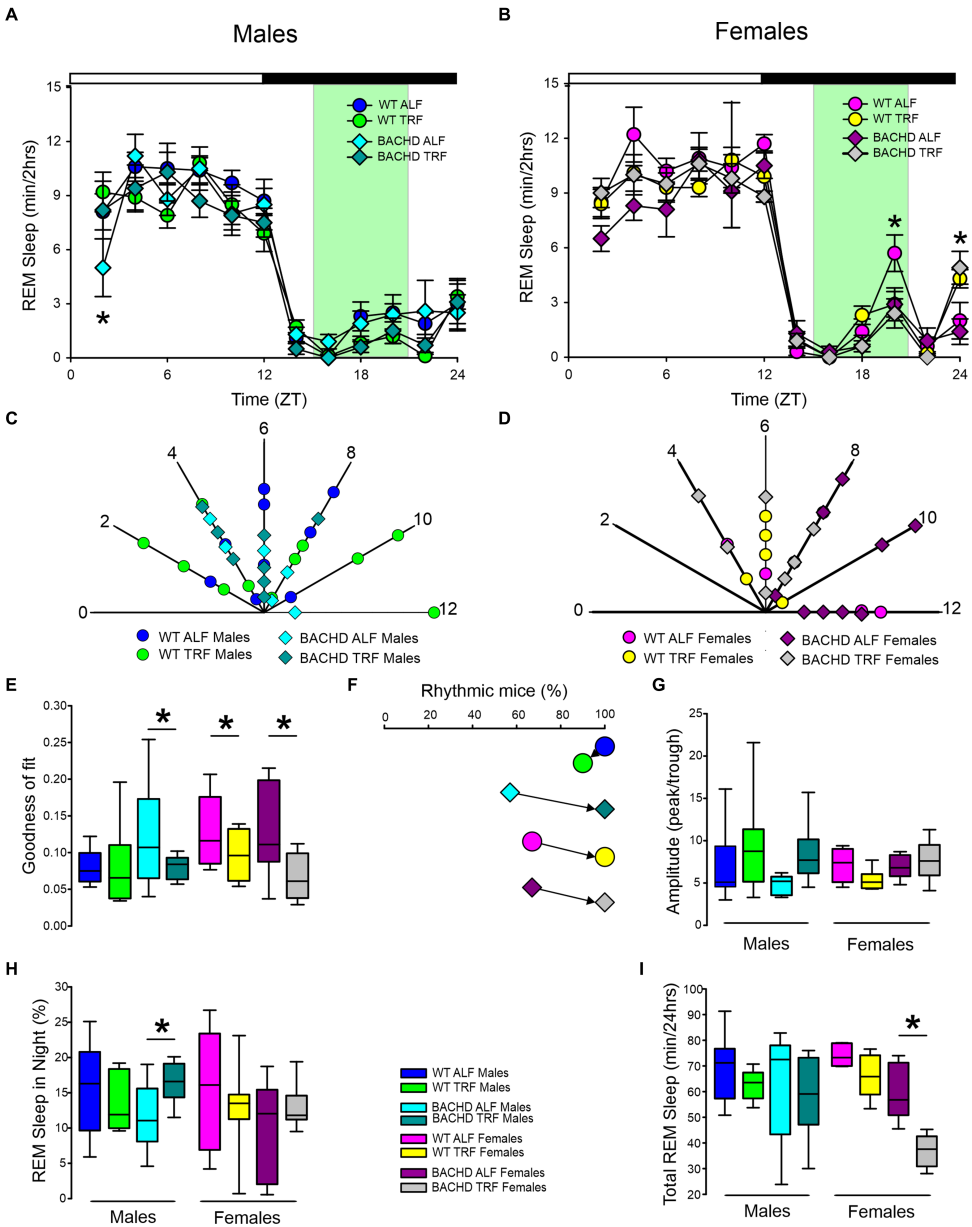
Temporal pattern of REM sleep in BACHD and WT mice under TRF and ALF. EEG recordings were conducted over 24-h in undisturbed mice held in a 12 h:12 h LD cycle. Waveforms showing the daily rhythms of REM sleep in male **(A)** and female **(B)** WT and BACHD mice plotted in 2-h bins. The BACHD males displayed reduced REM sleep at ZT 2 and this reduction was corrected by the TRF intervention. In WT females, the intervention reduced REM sleep at ZT 20. Data are presented as the mean ± SEM (*n* = 6–9 animals/sex/genotype/experimental group). Time points in which significant differences (*p* < 0.05) were found between the groups using multiple comparisons procedures (Holm-Sidak method) are indicated by one asterisk. Polar display of the peak phase of the rhythms of REM sleep in male **(C)** and female **(D)** WT and BACHD mice. The numbers on the axis represent zeitgeber time (ZT) with ZT 12 indicating the time of lights-off. There were not obvious differences among the groups in REM sleep phase. The “goodness of fit” was determined for the REM sleep measured from each animal **(E)**, the median calculated and shown in the box plot. Percentage of mice exhibiting a significant diurnal rhythm for each group **(F)**. The scheduled feeding increased the number of mice with improved rhythms in REM sleep in all groups except the WT males. The amplitude of the rhythms **(G)** as well as the percentage of REM sleep during the night **(H)** and the total minutes of recorded REM sleep **(I)** are shown for each group. TRF increased the percentage of REM sleep in the night in the male BACHD but reduced the total amount of REM sleep in the female BACHD. The box plots show the distribution of numerical data and skewness by displaying the data quartiles and median. *p < 0.05, Holm-Sidak multiple comparisons test.

Next, we carried out a similar analysis on the temporal patterns of REM sleep, and examined the rhythm in REM sleep looking at 2-h bins across the 24-h cycle under ALF and TRF (Figures 2A,B). We analyzed the waveforms using a three-way ANOVA with genotype, treatment, and time as factors for both male and female WT and mutant mice. Time was highly significant for both sexes. Treatment only exhibited significant effects in the male mice, while the females displayed significant differences by genotype (Table 1), along with a significant interaction between treatment and time. Both sex and the feeding schedule significantly affected the phase of REM sleep (Figures 2C,D; Table 2). We did not observe a large variability in the male BACHD by other measures, while the WT females exhibited the most variability in the peak phase (Table 3). The percentage of mice with a significant diurnal rhythm in REM sleep was increased by the treatment in all but the WT males (Figures 2E,F; Tables 4, 5). The amplitude of the rhythm in REM sleep was not was significantly affected by genotype, treatment, or sex (Tables 2, 5), as well as the total amount of REM sleep (Figures 2G–I; Table 2). Thus, TRF specifically altered the phase of the rhythm in REM sleep with the primary effect in females.

## NREM sleep

**Figure 3:**
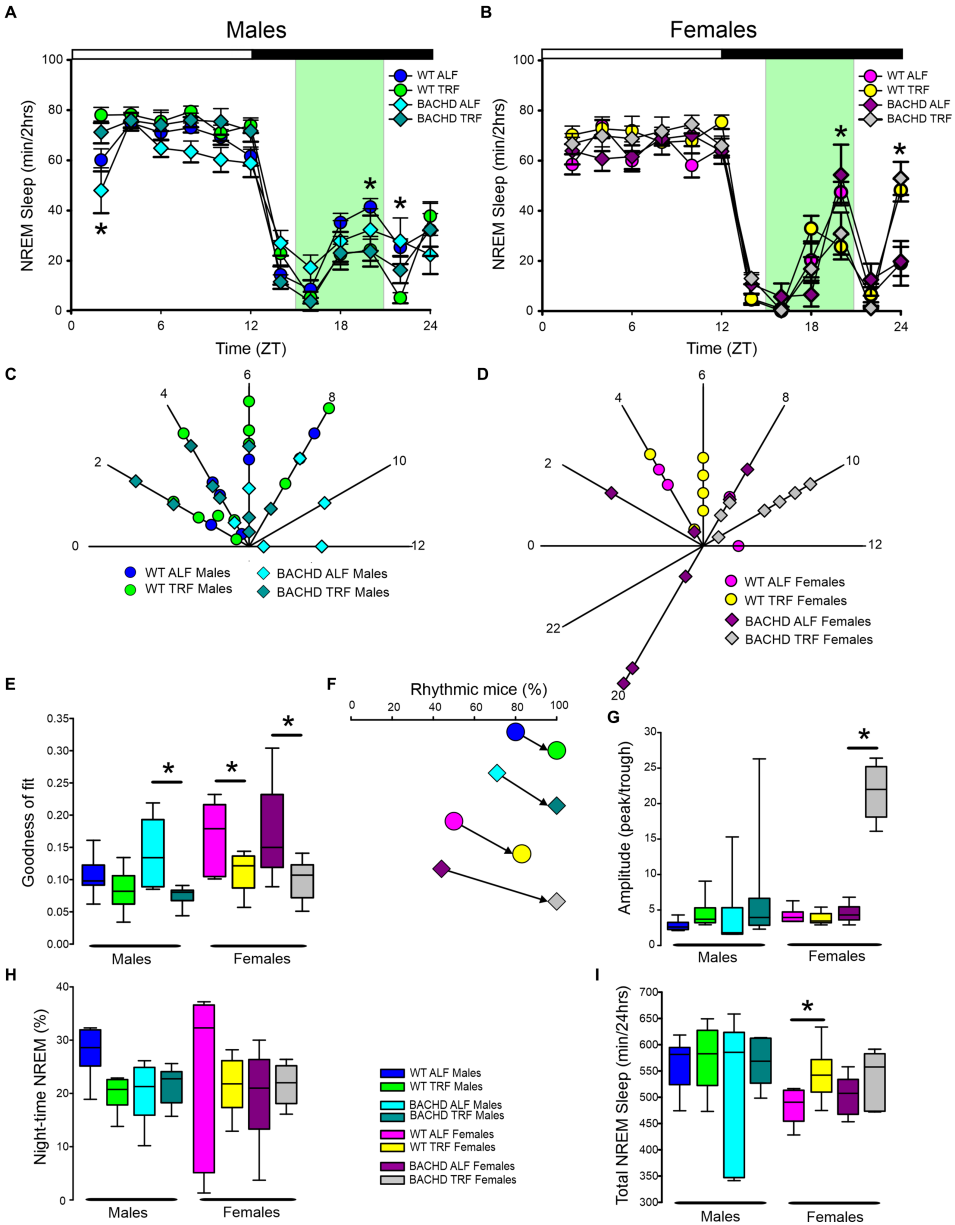
Temporal pattern of NREM sleep in BACHD and WT mice under TRF and ALF. EEG recordings were conducted over 24-h in undisturbed mice held in a 12 h:12 h light/dark cycle. Waveforms showing the daily rhythms of NREM sleep in male **(A)** and female **(B)** WT and BACHD mice plotted in 2-h bins. The BACHD males displayed reduced NREM sleep at ZT 2 and this reduction was corrected by the TRF intervention. In WT mice, the intervention reduced NREM sleep at ZT 20. Data are presented as the mean ± SEM (*n* = 6–9 animals/sex/genotype/experimental group). Time points in which significant differences (*p* < 0.05) were found between the groups using multiple comparisons procedures (Holm-Sidak method) are indicated by one asterisk. Polar display of the peak phase of the rhythms of REM sleep in male **(C)** and female **(D)** WT and BACHD mice. The female BACHD under ALF exhibited higher variability in peak phase than the other groups. The “goodness of fit” was determined for the NREM sleep measured from each animal **(E)**, the median calculated and shown in the box plot. Percentage of mice exhibiting a significant diurnal rhythm for each group plotted **(F)**. The amplitude of the rhythms **(G)** as well as the % of NREM sleep during the night **(H)** and the total minutes of recorded NREM sleep **(I)** are displayed for each group. The TRF treatment increased the amplitude and the total amount of NREM sleep in BACHD and WT female mice, respectively. The box plots show the distribution of numerical data and skewness by displaying the data quartiles and median. *p < 0.05, Holm-Sidak multiple comparisons test.

The rhythm in NREM sleep were analyzed in 2-h bins across the 24-h cycle in mice held on ALF and TRF (Figures 3A,B) and then using a three-way ANOVA with genotype, treatment, and time as factors for both male and female WT and BACHD mice. For both sexes, regardless of genotype or feeding regimen, time was highly significant along with a significant interaction between treatment and time (Table 1). Importantly, the reduction in NREM sleep at ZT 2 characteristic of the male BACHD was corrected by the TRF intervention (Figure 3A). Genotype had a significant effect on the peak phase of the NREM sleep rhythms (Figures 3C,D; Table 2). The BACHD exhibited a great deal of variability in phase (Table 3) compared to the WT, which was reduced by TRF. For NREM sleep, such effect was even more robust in the female BACHD. The percentage of mice with a significant diurnal rhythm in NREM sleep was increased by the treatment (Figures 3E,F; Table 4). Cosinor analysis showed an increase in the amplitude of the NREM rhythms and a significant effect of TRF, particularly evident in the BACHD females (Figure 3G; Table 5). On the other hand, the % sleep during the night did not vary with genotype, sex, or feeding schedule (Figures 3G,H; Table 2). The total amount of NREM sleep exhibited sex differences with an increase in the WT females on TRF (Figure 3I).

**Figure 4:**
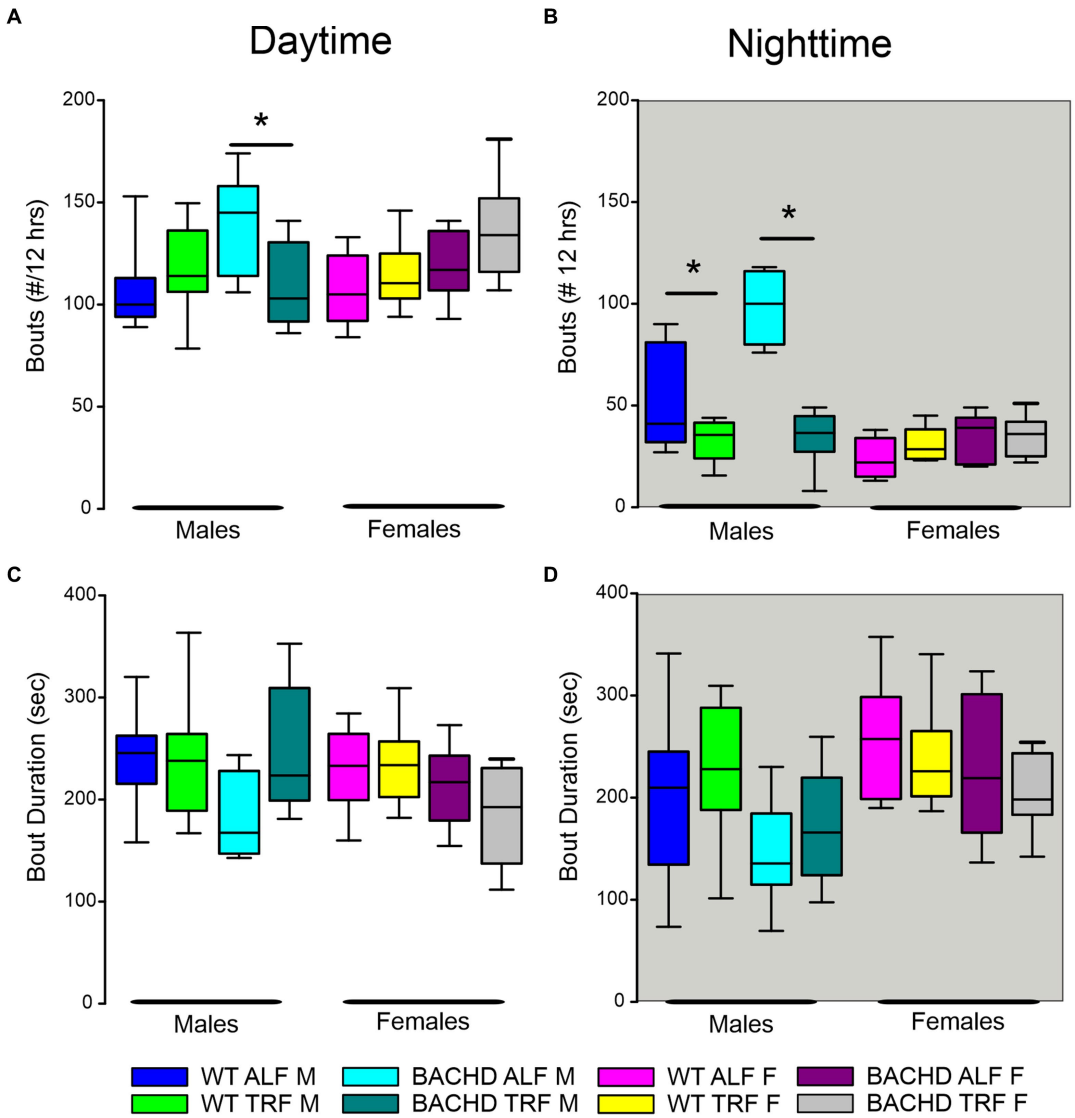
NREM sleep fragmentation in BACHD and WT mice under TRF and ALF. Sleep fragmentation during NREM sleep was calculated from the 24-h EEG recordings. The number and average duration of the bouts were measured during the **(A,C)** day and **(B,D)** night. In both the day and night, the male mutants exhibited an increased number of bouts **(A,B)** that was reduced to WT levels by the TRF intervention. No significant differences were seen with the bout duration. The box plots show the distribution of numerical data and skewness by displaying the data quartiles and median (*n* = 6–9 animals/sex/genotype/experimental group). *p < 0.05, Holm-Sidak multiple comparisons test.

Finally, we assessed sleep fragmentation by measuring the number of NREM sleep bouts and their duration using a three-way ANOVA with genotype, treatment, and sex as factors for day and night (Figure 4). During the day, there were significant effects of genotype but not treatment and sex, along with a significant interaction among the three factors (Table 6). Specifically, the male BACHD had more bouts than the WT and this difference was absent in mutants on the TRF regimen (Figure 4A). During the night, there were significant effects of genotype, treatment, and sex, as well as a significant interaction among these factors (Table 6). Again, the male BACHD were the most impacted and displayed a higher number of NREM sleep bouts of shorter duration. TRF reduced the number of bouts to WT levels in the mutant males.

**Table 6:** Analysis of NREM sleep fragmentation by three-way ANOVA with genotype, sex, and treatment as factors.

NREM		Genotype	Sex	Treatment	Interaction Genotype x Sex x Time
Bout #	Day	***F***_**(1,62)**_ **= 7.306, *p* = 0.009**	*F*_(1,62)_ = 0.032, *p* = 0.857	*F*_(1,62)_ = 0.062, *p* = 0.805	***F***_**(1,62)**_ **= 5.616, *p* = 0.021**
	Night	***F***_**(1,62)**_ **= 15.10, *p* < 0.001**	***F***_**(1,62)**_ **= 41.542, *p* < 0.001**	***F***_**(1,62)**_ **= 26.77, *p* < 0.001**	***F***_**(1,62)**_ **= 6.361, *p* = 0.015**
Bout duration	Day	***F***_**(1,62)**_ **= 5.893, *p* = 0.019**	*F*_(1,62)_ = 1.211, *p* = 0.276	*F*_(1,62)_ = 0.768, *p* = 0.385	***F***_**(1,62)**_ **= 4.652, *p* = 0.035**
	Night	***F***_**(1,62)**_ **= 7.101, *p* = 0.010**	***F***_**(1,62)**_ **= 9.156, *p* = 0.004**	*F*_(1,62)_ = 0.051, *p* = 0.822	*F*_(1,62)_ = 0.005, *p* = 0.914

Interactions among the three factors are reported. Degrees of freedom are reported within parentheses, alpha = 0.05. Bold type indicates statistical significance.

### EEG spectral power

**Figure 5:**
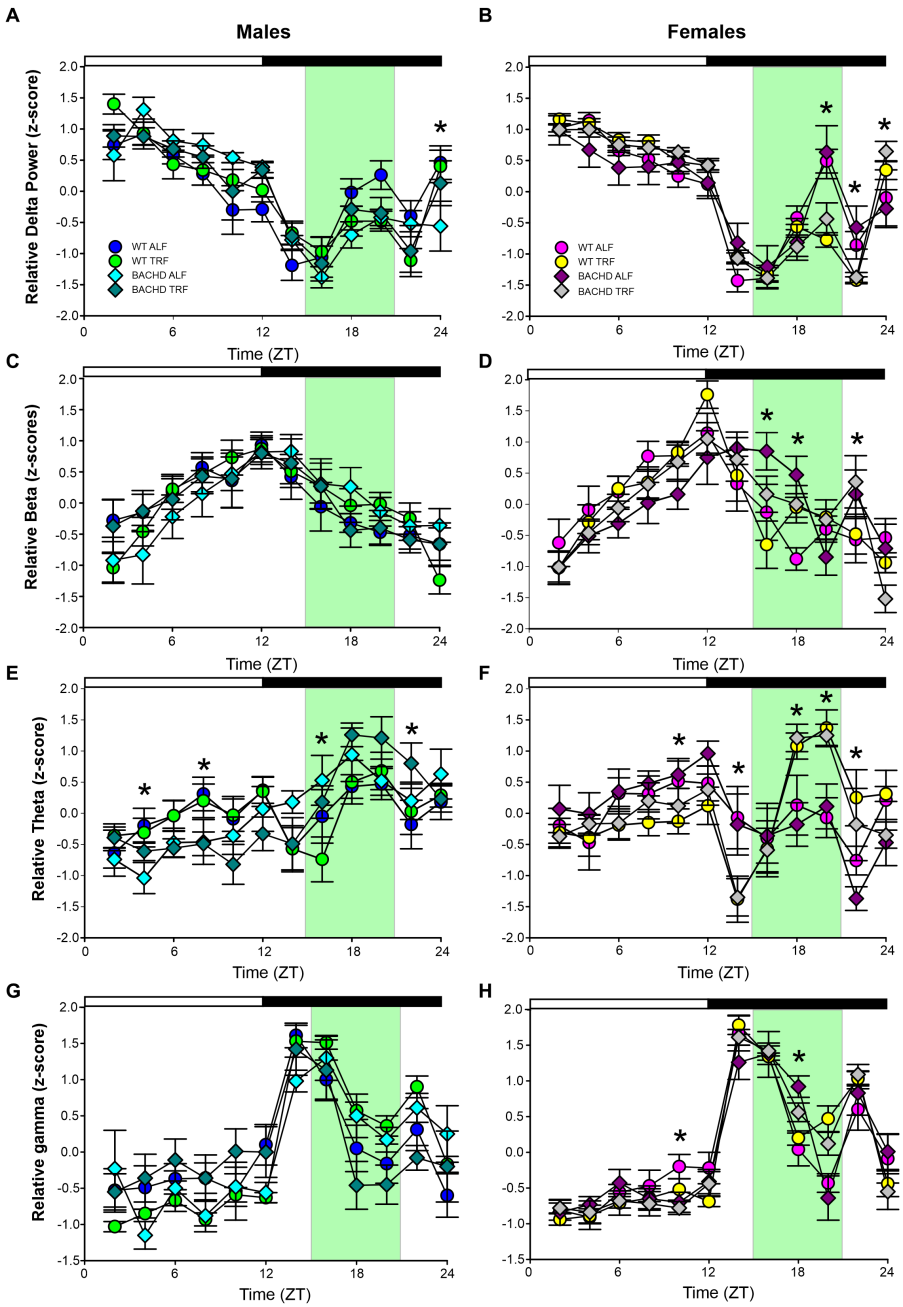
Absolute and relative power spectral analysis in BACHD and WT mice under TRF and ALF. Power spectral analysis was performed by applying a fast Fourier transform to raw 24-h EEG waveforms. The relative power for delta **(A,B)**, beta **(C,D)**, theta **(E,F)** and gamma **(G,H)** power was also plotted for each group. The green shading indicates the time of feeding for the TRF groups. The symbols shown in panels A and B apply to the rest of the figure. Data are shown as the mean ± SEM of 6–9 animals/sex/genotype/experimental group. Frequencies in which treatment-evoked significant differences (*p* < 0.05) were found with between the groups using multiple comparison procedures (Holm-Sidak method) are indicated by one asterisk. The theta rhythm was most impacted by the scheduled feeding.

Young BACHD mice exhibit modest changes in the EEG spectra compared to WT mice with sex differences also noted (Chiem et al., 2024). To investigate the possible impact of TRF on the EEG spectra, we quantified the power values in the frontoparietal cortical region during NREM sleep. Analysis of the power spectral curves (0.1-40 Hz) with LME model found evidence for significant differences of frequency, genotype, and sex (Table 7). We also used a three-way ANOVA to probe for possible impacts of the diet (Table 8). Treatment affected the power spectral curves for male BACHD only during the day, by primarily altering the lower end of the frequency spectrum. We also examined the relative power throughout the 24-h cycle for delta (0.5–4 Hz), theta (5–7 Hz), beta (14–20 Hz), and gamma (30–40 Hz) (Figures 5A–H). The LME model found significant effects of time for delta, theta and gamma (Table 7). This analysis strikingly highlighted the broad impact of all the factors on theta rhythms. Time exhibited a significant effect on all the frequency bands and a significant interaction between time and treatment was observed in the females, as determined by three-way ANOVA (Table 9). These interactions were statistically robust and suggest a complexity in the female response to the TRF regimen. Overall, apart from theta rhythms, the analysis of the spectral distribution of the EEG did not uncover robust impacts of the scheduled feeding.

**Table 7:** Analysis of absolute and relative power (z-score) using a linear mixed effects (LME) model.

Absolute power	Estimate (SE)	df	*t*	*p*
Intercept	**15537.554 (80.872)**	**316**	**19.012**	**< 0.001**
Hz	**−64.460 (3.582)**	**316**	**−17.995**	**< 0.001**
Genotype	**−302.450 (123.534)**	**316**	**-2.448**	**0.014**
Sex	**−570.009 (123.534)**	**316**	**-4.614**	**< 0.001**
treatment	25.892 (118.385)	316	0.219	0.827
**Delta**	Estimate (SE)	df	*t*	*p*
Intercept	**0.864 (0.167)**	**764**	**5.070**	**< 0.001**
Genotype	0.124 (0.252)	764	0.492	0.623
Sex	0.328 (0.252)	764	1.300	0.194
time	**−0.065 (0.011)**	**764**	**−5.742**	**< 0.001**
treatment	0.269 (0.252)	764	1.068	0.286
**Beta**	Estimate (SE)	df	*t*	*p*
Intercept	−0.087 (0.195)	764	−0.445	0.656
Genotype	0.530 (0.296)	764	1.789	0.074
Sex	−0.159 (0.296)	764	−0.538	0.591
time	0.006 (0.013)	764	0.504	0.615
treatment	0.158 (0.296)	764	0.534	0.593
**Theta**	Estimate (SE)	df	*t*	*p*
Intercept	**0.680 (0.187)**	**764**	**3.634**	**< 0.001**
Genotype	**−0.564 (0.283)**	**764**	**−1.994**	**0.046**
Sex	**−1.654 (0.283)**	**764**	**−5.845**	**< 0.001**
time	**−0.052 (0.012)**	**764**	**−4.115**	**< 0.001**
treatment	**−0.936 (0.283)**	**764**	**−3.308**	**< 0.001**
**Gamma**	Estimate (SE)	df	*t*	*p*
Intercept	**−0.895 (0.177)**	**650**	**−5.068**	**< 0.001**
Genotype	0.174 (0.267)	650	0.672	0.502
Sex	0.006 (0.279)	650	−0.023	0.981
time	**0.007 (0.001)**	**650**	**5.739**	**< 0.001**
treatment	0.002 (0.267)	650	-0.099	0.921

The LME model incorporate fixed effects (genotype, treatment, sex, and time) as well as helping us understand the extent to which variability in the outcome is explained by the fixed and random effects. This analysis was not used for hypothesis testing. Bold type indicates statistical significance.

**Table 8:** Analysis of power (absolute) spectral curves during the day and the night by three-way ANOVA with genotype, sex, and frequency (1-40 Hz) as factors.

		Genotype	Frequency (Hz)	Treatment
Males	Day	***F***_**(1,809)**_ **= 12.296, *p* < 0.001**	***F***_**(29,809)**_ **= 90.26, *p* < 0.001**	***F***_**(1,809)**_ **= 16.93, *p* < 0.001**
	Night	*F*_(1,809)_ = 1.129, *p* = 0.288	***F***_**(29,809)**_ **= 26.91, *p* < 0.001**	*F*_(1,809)_ = 0.806, *p* = 0.370
Females	Day	***F***_**(1,809)**_ **= 28.400, *p* < 0.001**	***F***_**(29,809)**_ **= 96.00, *p* < 0.001**	*F*_(1,809)_ = 0.808, *p* = 0.369
	Night	***F***_**(1,809)**_ **= 7.498, *p* = 0.006**	***F***_**(29,809)**_ **= 49.52, *p* < 0.001**	*F*_(1,809)_ = 0.156, *p* = 0.693

No significant interactions among the three factors were detected. Degrees of freedom are reported within parentheses, alpha = 0.05. Bold type indicates statistical significance.

**Table 9:** Analysis of relative power (z-score) spectral curves during the day and the night by three-way ANOVA with genotype, treatment, and time as factors.

Frequency	Sex	Genotype	Time (2 h bins)	Treatment	Interaction Treatment x Time
Delta	M	*F*_(1,431)_ < 0.001, *p* = 0.995	***F***_**(11,431)**_ **= 35.36, *p* < 0.001**	*F*_(1,431)_ < 0.001, *p* = 0.999	*F*_(11,31)_ = 2.207, *p* = 0.089
	F	*F*_(1,347)_ < 0.001, *p* = 0.996	***F***_**(11,347)**_ **= 75.71, *p* < 0.001**	*F*_(1,347)_ < 0.001, *p* = 0.994	***F***_**(11,347)**_ **= 6.005, *p* < 0.001**
Beta	M	*F*_(1,431)_ < 0.001, *p* = 0.999	***F***_**(11,431)**_ **= 13.18, *p* < 0.001**	*F*_(1,431)_ < 0.001, *p* = 1.0	*F*_(11,31)_ = 0.420, *p* = 0.947
	F	*F*_(1,347)_ < 0.001, *p* = 0.998	***F***_**(11,347)**_ **= 21.47, *p* < 0.001**	*F*_(1,347)_ < 0.001, *p* = 0.996	***F***_**(11,335)**_ **= 2.755, *p* = 0.002**
Theta	M	*F*_(1,431)_ < 0.001, *p* = 0.997	***F***_**(11,419)**_ **= 9.471, *p* < 0.001**	*F*_(1,431)_ < 0.001, *p* = 1.0	*F*_(11,31)_ = 1.105, *p* = 0.356
	F	*F*_(1,347)_ < 0.001, *p* = 0.999	***F***_**(11,347)**_ **= 8.837, *p* < 0.001**	*F*_(1,347)_ < 0.001, *p* = 0.998	***F***_**(11,335)**_ **= 7.405, *p* < 0.001**
Gamma	M	*F*_(1,431)_ < 0.001, *p* = 0.999	***F***_**(11,419)**_ **= 30.13, *p* < 0.001**	*F*_(1,431)_ < 0.001, *p* = 1.0	*F*_(11,31)_ = 0.546, *p* = 0.871
	F	*F*_(1,347)_ < 0.001, *p* = 0.999	***F***_**(11,347)**_ **= 84.56, *p* < 0.001**	*F*_(1,347)_ < 0.001, *p* = 0.998	***F***_**(11,335)**_ **= 2.999, *p* = 0.001**

Interactions between the time and treatment are reported. Degrees of freedom are reported within parentheses, alpha = 0.05. Bold type indicates statistical significance. M = males; F = females.

## Recovery from sleep deprivation

**Figure 6:**
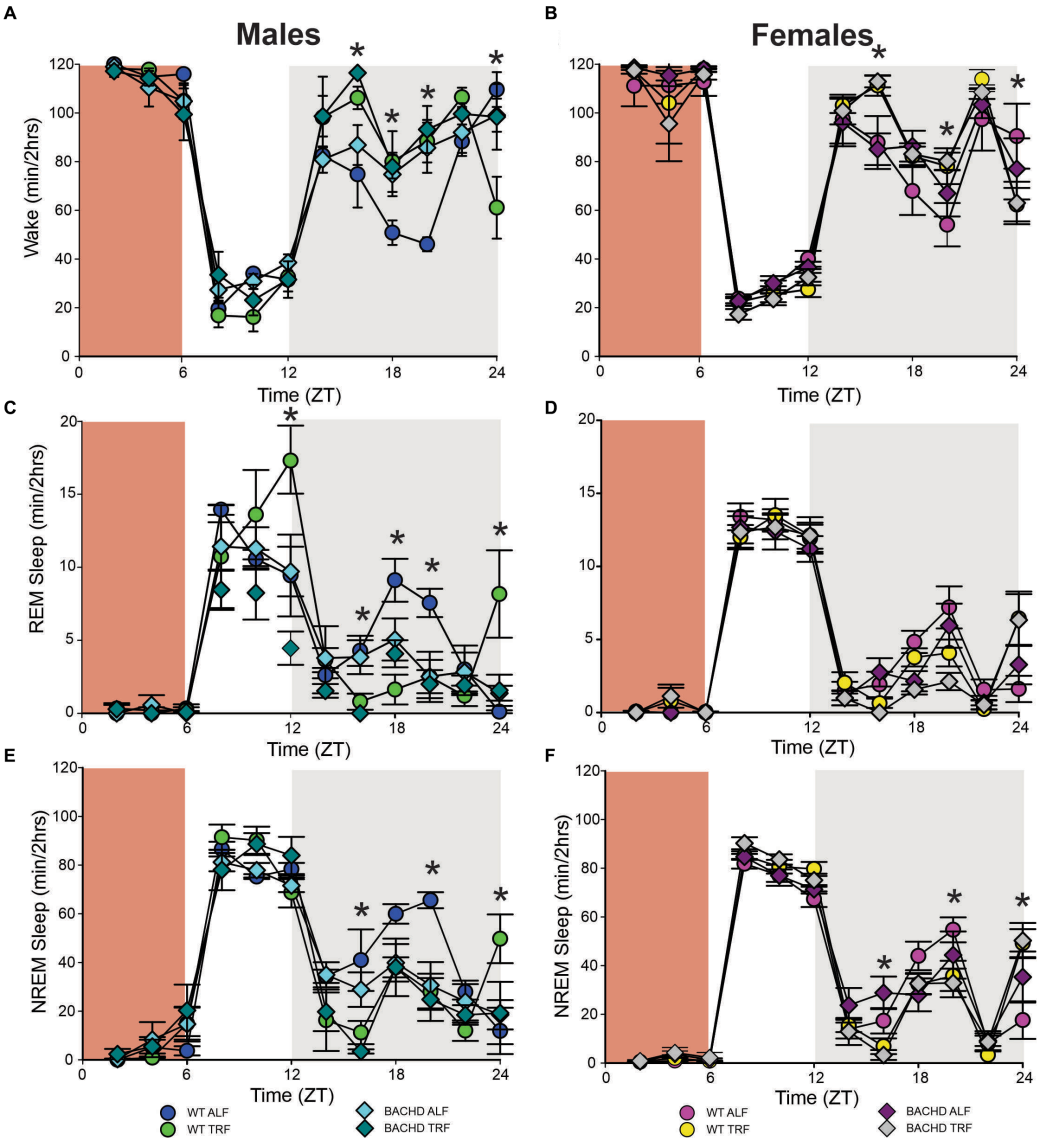
Sleep homeostatic mechanisms in BACHD mice. Mice were exposed to a 6-h sleep deprivation (SD) at the beginning of their inactive phase (ZT0-6) using a gentle-handling protocol. EEG recordings were conducted over 24-h. Waveforms showing the daily rhythms in wake **(A,B)**, REM sleep **(C,D)**, and NREM sleep **(E,F)** are plotted in 2-h bins. The red-shaded area represents the time of SD. All the groups showed a strong increase in sleep between ZT6 and ZT12. Data are shown as the mean ± SEM of 6–9 animals/sex/genotype/experimental group. Time points in which significant differences (*p* < 0.05) were found between the groups using multiple comparisons procedures (Holm-Sidak method) are indicated by one asterisk.

The most direct test of sleep homeostatic mechanisms is to examine sleep rebound in response to SD. Therefore, we examined the 18-h of recovery interval following 6-h of SD from ZT 0–6. The analysis of the waveforms for each sleep state (Figures 6A–F) was perfomed by three-way ANOVA with genotype, treatment, and time (2-h bins) as factors (Table 10). The SD protocol was equally successful in all groups, all of which exhibited a sleep rebound after SD (Figures 6A–F) and time was a robustly statistically significant factor (Table 10). We detected genotypic differences for wake in males and for REM sleep in females. Treatment did not produce any significant impact on the recovery, although we did see a significant interaction between treatment and time that indicates a complexity to the sleep deprivation response (Table 10). Analysis of recovery sleep between ZT 6–12, showed a strong increase in the amount of sleep in all the groups (Figure 6). Interestingly, the groups on the TRF regimen exhibited more NREM sleep [*F*_(3,46)_ = 6.181, *p* = 0.001, Figures 6E,F] regardless of sex or genotype. To account for potential baseline differences in NREM sleep, we analyzed the proportion of sleep gained during recovery relative to sleep lost during SD, but found no differences among the groups (Table 11). When the normalized relative NREM delta power was averaged across the recovery period (ZT6-24) for each animal, only a significant effect of sex was observed in the response (Table 11). Overall, the sleep homeostatic process appeared to be functional in all groups with relatively small differences observed.

**Table 10:** Analysis of waveforms in response to sleep deprivation (SD) using three-way ANOVA with genotype, time (2 h bins), and treatment as factors.

State	Sex	Genotype	Time(2 h bins)	Treatment	Interaction Treatment x Time
Wake	M	***F***_**(1,239)**_ **= 5.406, *p* = 0.021**	***F***_**(11, 239)**_ **= 75.96, *p* < 0.001**	*F*_(1, **239**)_ = 4.113, *p* = 0.044	***F***_**(11,239)**_ **= 4.561, *p* < 0.001**
	F	*F*_(1,320)_ = 0.068, *p* = 0.795	***F***_**(11,320)**_ **= 120.6, *p* < 0.001**	*F*_(1,320)_ = 0.002, *p* = 0.961	***F***_**(11,320)**_ **= 3.840, *p* < 0.001**
REM	M	*F*_(1,239)_ = 1.141, *p* = 0.286	***F***_**(11,239)**_ **= 21.39, *p* < 0.001**	*F*_(1,239)_ = 2.979, *p* = 0.086	*F*_(11,239)_ = 1.746, *p* = 0.066
	F	***F***_**(1,320)**_ **= 5.004, *p* = 0.026**	***F***_**(11,320)**_ **= 160.2, *p* < 0.001**	*F*_(1,330)_ = 0.173, *p* = 0.678	***F***_**(11,320)**_ **= 4.415, *p* < 0.001**
NREM	M	*F*_(1,239)_ = 3.209, *p* = 0.075	***F***_**(11,239)**_ **= 71.70, *p* < 0.001**	*F*_(1,239)_ = 3.398, *p* = 0.067	***F***_**(11,419)**_ **= 4.209, *p* < 0.001**
	F	*F*_(1,320)_ = 0.561, *p* = 0.455	***F***_**(11,320)**_ **= 201.6, *p* < 0.001**	*F*_(1,320)_ = 0.005, *p* = 0.940	***F***_**(11,320)**_ **= 5.238, *p* < 0.001**

Interactions between the time and treatment are reported. Degrees of freedom are reported within parentheses, alpha = 0.05. Bold type indicates statistical significance. M = males; F = females.

**Table 11:** Analysis of the response to SD by three-way ANOVA with genotype, sex, and treatment as factors.

NREM	Genotype	Sex	Treatment
NREM sleep gained/loss	*F*_(1,48)_ = 0.107, *p* = 0.745	*F*_(1,48)_ = 0.261, *p* = 0.612	*F*_(1,48)_ = 1.536, *p* = 0.222
rDelta/NREM	*F*_(1,48)_ = 0.172, *p* = 0.680	***F***_**(1,48)**_ **= 14.770, *p* < 0.001**	*F*_(1,48)_ = 0.191, *p* = 0.664

No significant interactions among the three factors were detected. Degrees of freedom are reported within parentheses, alpha = 0.05. Bold type indicates statistical significance. Relative Delta power (rDelta) refers to the proportion of delta wave activity within a specific frequency band relative to the total power of all frequency bands in an EEG signal.

## Discussion

Several good models of HD have been created, each with their own advantages and disadvantages (Pouladi et al., 2013). To explore the intersection between circadian dysfunction and neurodegenerative disorders, we have been working primarily with the BACHD mouse model of HD. This model expresses the full-length human mutant Htt (mHtt) with 97 CAG repeats (Gray et al., 2008) and has strong construct validity as it expresses the human mutation under the control of the gene’s endogenous promoter. The face validity is also high as the BACHD model has been shown to reproduce progressive behavioral deficits such as sleep and circadian disturbances (Kudo et al., 2011; Kuljis et al., 2016, 2018; Chiem et al., 2024) as well as selective cortical and striatal atrophies (Gray et al., 2008; Menalled et al., 2009). Of course, no animal model is perfect and the BACHD mice gain body weight with age (Pouladi et al., 2012), whereas HD patients exhibit weight loss. Also, these mutants do not express the same transcriptional signature seen in other HD models (Gu et al., 2022). Notwithstanding these shortcomings, we feel that this mutant provides a model with strong construct and face validity. In a series of studies, we have detailed the progression of sleep, circadian, and cardiovascular deficits along with sex differences in the BACHD model (Kudo et al., 2011; Kuljis et al., 2016, 2018; Schroeder et al., 2016; Park et al., 2021; Chiem et al., 2024), which encouraged our use of this model for circadian-based interventions.

One of the most powerful regulators of the circadian system is the daily feed/fast cycle and many studies have found benefits in scheduled feeding (Acosta-Rodríguez et al., 2022; Hepler et al., 2022; Mihaylova et al., 2023). In the present study, we used EEG to determine if scheduled feeding countered HD-driven changes in the temporal patterning of vigilance states (wake, NREM sleep, REM sleep) in the BACHD model in 6-month-old mice. We exposed the mice at the onset of their symptoms to a 6-h feeding/18-h fasting regimen aligned with the middle (ZT 15–21) of their active time (ZT 12–24). Following 1 month of treatment, we found that TRF altered several key sleep parameters with the BACHD males being particularly responsive. The scheduled feeding reduced inappropriate activity at the beginning of the day and increased the NREM sleep at the same phases. The TRF notability reduced variability in the peak phase of wake and NREM sleep in the mutants. The number of sleep bouts was notably reduced in the TRF treated mice. Our present findings demonstrate clear dysfunction in the sleep/wake architecture and sleep fragmentation in adult male BACHD mice compared to WT controls and that these deficits were improved by TRF treatment.

These new findings fit into a body of work where we tested whether TRF improved symptoms of HD in the BACHD (Whittaker et al., 2018) and Q175 (Wang et al., 2018) mouse models. In prior work, we demonstrated that the mutants treated with TRF for three months showed improvements in locomotor activity and sleep behavioral rhythms, as well as in heart rate variability, suggesting an amelioration of the autonomic nervous system dysfunction. In prior work, we demonstrated that TRF altered the phase of the rhythms of the clock gene PER2 measured both *in vivo* and *in vitro* (Whittaker et al., 2018). Importantly, TRF-treated BACHD (Whittaker et al., 2018) and Q175 (Wang et al., 2018) models exhibited improved motor performance compared with untreated mutants, and the motor improvements were correlated with improved circadian output. In the Q175 line, we found that the expression of several HD-relevant markers was restored to WT levels in the striatum of the treated mice using NanoString gene expression assays including BDNF signaling pathways (Wang et al., 2018).

In this earlier work, we documented TRF improvements in sleep behavior. These behavioral measures of sleep are widely used by the *Drosophila* research community (e.g., Artiushin and Sehgal, 2017) and have found acceptance by behavioral neuroscientists as well (Pack et al., 2007; Fisher et al., 2012). Using behavioral measures of sleep, we have been able to show that three months of TRF improved the precision of sleep onset while the fragmentation of sleep was reduced (Wang et al., 2018; Whittaker et al., 2018). There has been a report in *Drosophila* that TRF can improve sleep behavior (Gill et al., 2015). While behavioral measures are powerful, there is a concern of using activity measures in models that are known to exhibit motor dysfunction. In rodent models, EEG determinants of sleep help address this concern about activity measures.

The possibility of sex differences in the HD phenotype has been a prominent feature of our earlier work. Data from mouse models including the CAG 140 (Dorner et al., 2007) and Q175 (Padovan-Neto et al., 2019), as well as the BACHD mouse model (Kuljis et al., 2016), all suggest that males are more impacted early in the disease trajectory. In recent work, we also documented sex differences in the EEG-defined sleep/wake cycle of BACHD mice at 3-months of age (Chiem et al., 2024). We found male BACHD mice exhibited reduced amounts of NREM sleep in the day, a feature which is not seen in the female BACHD. Additionally, the male BACHD mice exhibited a striking increase in variability in the phase of the rhythms in each state. While not the focus of the present study, we continued to find sex differences in vigilance states of the sleep/wake cycle of BACHD mice at 4 months of age. Overall, the EEG measures of state are consistent with our prior behavioral data indicating the male BACHD mice are more impacted than female BACHD mice. Interestingly, recent analysis with HD patients also found evidence for a sex difference in disease trajectory with females being more vulnerable (Zielonka and Stawinska-Witoszynska, 2020; Hentosh et al., 2021). Clearly more work is needed but both human and animal research does raise the possibility that sex-specific factors play a role in the HD symptom progression.

EEG signals are widely used to evaluate function and dysfunction in the central nervous system and the functional importance of specific frequency bands is an area of active research (Saby and Marshall, 2012; Ibarra-Lecue et al., 2022; Andrillon and Oudiette, 2023). Several studies have found characteristic changes in the EEG spectra in mouse models including the R6/2 (Fisher et al., 2013; Kantor et al., 2013), the R6/1 (Lebreton et al., 2015), and Q175 (Fisher et al., 2016). When we examined absolute power, we saw clear sex differences in the lower end of the power spectrum, with BACHD females exhibiting increased power compared to the males. Statistically significant effects on genotype were seen in the males during the day and females in day and night. The TRF did produce significant changes in the males during the day but not in the other groups. Still, counter to our expectations, we did not see any impact of the diet on the individual frequency bands with the ANOVAs. The linear mixed effects (LME) model did detect highly significant effects on the theta band. Given the association of hippocampal theta rhythms with memory formation and navigation, these TRF driven changes should be explored in future studies.

To test sleep homeostatic mechanisms, we examined how the BACHD and WT mice responded to a 6-h SD protocol. All groups were effectively sleep deprived and all groups responded to SD with an increase in sleep amount. There were no differences in the amount of sleep gained during recovery relative to sleep lost during SD or in the normalized relative NREM delta power during the recovery period. Scheduled feeding alone did not produce any significant impact on the recovery. We did see a significant interaction between treatment and time that indicates a complexity to the sleep deprivation response that would require additional experiments to clarify.

The mechanisms underlying these benefits of TRF on the central nervous system function are not known although there are several possibilities (Longo and Panda, 2016; Jensen et al., 2020; Bass, 2024). In recent work, we reported that a ketogenic diet (KD) ameliorates the symptoms and delays disease progression in the BACHD model (Whittaker et al., 2022). Mutant mice fed a KD for three months displayed increased daytime sleep and improved timing of sleep onset. In addition, KD improved activity rhythms and motor performance on the rotarod and challenging beam tests. Prior work has also shown benefits of the KD in the R6/2 model in reducing weight loss and improving open field behavior (Chen et al., 2016). It is noteworthy that the probiotic *Akkermansia municiphila* (*Akk*) dramatically increased in abundance under KD, since it has been associated with improved metabolic health and lower inflammation (Attaye et al., 2021; Bonnechère et al., 2022). Intriguingly, work in a *Drosophila* model of HD implicated the gut bacteria in the regulation of HD pathology (Chongtham et al., 2022), and diet-driven changes in the microbiome could also influence HD-pathology in the fly. In a project with the Desplats’s group, we recently exposed Alzheimer’s disease mouse models to the TRF protocol and reported that this regimen had the remarkable capability of simultaneously reducing amyloid deposition, increasing Aβ42 clearance, and normalizing daily transcription patterns of multiple genes (Whittaker et al., 2023). Together, these findings suggest that feeding schedule regimen and diet could play an important role in the development of new treatment options for HD and other neurodegenerative disorders.

## Data availability statement

The datasets presented in this study can be found in online repositories. The names of the repository/repositories and accession number(s) can be found in the article/supplementary material.

## Ethics statement

The animal study was approved by Animal Research Committee (ARC). The study was conducted in accordance with the local legislation and institutional requirements.

## Author contributions

EC: Data curation, Formal analysis, Investigation, Methodology, Software, Writing – original draft, Writing – review & editing. KZ: Investigation, Methodology, Writing – original draft, Writing – review & editing. DD’A: Formal analysis, Software, Writing – original draft, Writing – review & editing. CG: Project administration, Writing – original draft, Writing – review & editing. KP: Conceptualization, Funding acquisition, Supervision, Writing – original draft, Writing – review & editing. CC: Conceptualization, Data curation, Formal analysis, Funding acquisition, Project administration, Supervision, Writing – original draft, Writing – review & editing.

## Footnotes

^1^ https://www.circadian.org/softwar.html
